# Unravelling the genetic basis of retinal dystrophies in Pakistani consanguineous families

**DOI:** 10.1186/s12886-023-02948-8

**Published:** 2023-05-10

**Authors:** Muhammad Marwan, Muhammad Dawood, Mukhtar Ullah, Irfan Ullah Shah, Niamat Khan, Muhammad Taimur Hassan, Muhammad Karam, Lettie E. Rawlins, Emma L Baple, Andrew H. Crosby, Shamim Saleha

**Affiliations:** 1 Department of Biotechnology and Genetic Engineering, Kohat University of Science and Technology, Kohat, Khyber Pakhtunkhwa 26000 Pakistan; 2 Institute of Molecular and Clinical Ophthalmology Basel, Basel, 4031 Switzerland; 3 Department of Ophthalmology, University of Basel, Basel, 4056 Switzerland; 4 Department of Ophthalmology, KMU Institute of Medical Sciences KIMS, Kohat, Khyber Pakhtunkhwa 26000 Pakistan; 5 Medical Research, RILD Wellcome Wolfson Centre (Level 4), Royal Devon and Exeter NHS Foundation Trust, Exeter, Devon, EX2 5DW UK; 6 Peninsula Clinical Genetics Service, Royal Devon & Exeter Hospital (Heavitree), Exeter, UK

**Keywords:** Retinitis pigmentosa, Usher syndrome, Pakistani, *PED6C*, *MYO7A*, *RHO*, *MERTK*, *TULP1*.

## Abstract

**Background:**

Retinitis Pigmentosa (RP) is a clinically and genetically progressive retinal dystrophy associated with severe visual impairments and sometimes blindness, the most common syndromic form of which is Usher syndrome (USH). This study aimed to further increase understanding of the spectrum of RP in the Khyber Pakhtunkhwa region of Pakistan.

**Methodology:**

Four consanguineous families of Pashtun ethnic group were investigated which were referred by the local collaborating ophthalmologists. In total 42 individuals in four families were recruited and investigated using whole exome and dideoxy sequencing. Among them, 20 were affected individuals including 6 in both family 1 and 2, 5 in family 3 and 3 in family 4.

**Result:**

Pathogenic gene variants were identified in all four families, including two in cone dystrophy and RP genes in the same family (*PDE6C;* c.480delG, p.Asn161ThrfsTer33 and *TULP1;* c.238 C > T, p.Gln80Ter) with double-homozygous individuals presenting with more severe disease. Other pathogenic variants were identified in *MERTK* (c.2194C > T, p.Arg732Ter), *RHO* (c.448G > A, p.Glu150Lys) associated with non-syndromic RP, and *MYO7A* (c.487G > A, p.Gly163Arg) associated with USH. In addition, the reported variants were of clinical significance as the *PDE6C* variant was detected novel, whereas *TULP1*, *MERTK*, and *MYO7A* variants were detected rare and first time found segregating with retinal dystrophies in Pakistani consanguineous families.

**Conclusions:**

This study increases knowledge of the genetic basis of retinal dystrophies in families from Pakistan providing information important for genetic testing and diagnostic provision particularly from the Khyber Pakhtunkhwa region.

**Supplementary Information:**

The online version contains supplementary material available at 10.1186/s12886-023-02948-8.

## Introduction

Retinitis pigmentosa (RP) is an inherited retinal dystrophy, affecting 1 in 4000 individuals [1]. RP is caused by the progressive degeneration of photoreceptors cells in the retina and characterized by absence or scarcity of retinal pigment deposits visible on fundus examination. Initially, degeneration of rod photoreceptors results in night blindness and then progressive loss of peripheral vision leads to tunnel vision. Disease progression results in decreased visual acuity due to cone photoreceptors degeneration that eventually leads to complete blindness [2]. It is generally considered that about 70-80% of all RP cases are non-syndromic, where eyes are exclusively affected [3]. However, the most common syndromic form of RP is Usher syndrome (USH), characterized by sensorineural hearing loss, RP, and, in some cases, vestibular dysfunction [4]. USH is the most frequent cause of deaf-blindness and affects more than 50% of both deaf and blind individuals in different populations [5]. Importantly, the age of onset, severity of symptoms and disease progression of both RP and USH may vary widely among patients, even within the same family [6–9].

RP manifests almost all patterns of inheritance, that is an autosomal dominant, autosomal recessive, X-linked, including rare non-Mendelian forms such as mitochondrial or digenic inheritance patterns [10, 11]. About 60% of RP cases are inherited in autosomal recessive manner [6]. In addition, the USH represents about 10–30% of all autosomal recessive RP (arRP) cases [5]. Although, RP shows no ethnic specificity, however, the autosomal recessive forms of RP have been more frequently reported in Chinese, Israeli, Saudi, Turkey, south Indian and Pakistani populations which are geographically isolated and display relatively high consanguinity rates [12–16]. Importantly, studying patients with phenotypes of recessively inherited diseases like RP and USH in geographically isolated and highly consanguineous populations may be useful to identify disease causal mutations [17].

RP has great phenotypic and genetic heterogeneity due to many associated genetic defects, each of which has numerous alleles and corresponds to a gene or locus-specific phenotype [16]. Over 130 genes have been found to be associated with RP (http://www.sph.uth.tmc.edu/RetNet), including approximately 90 genes for non-syndromic RP and about 40 genes for syndromic forms [18]. Genes commonly reported to cause RP include *ABCA4* (MIM #601691), *CRB1* (MIM #604210), *CDHR1* (MIM #609502), *PDE6A* (MIM #180071), *PDE6B* (MIM #180072), *RHO* (MIM #180380), *RP1* (MIM #603937), *SPATA7* (MIM #609868), *EYS* (MIM #612424), *RP1L1* (MIM #608581), *TULP1* (MIM #602280), and *USH2A* (MIM #608400). Similarly, the commonly reported USH causative genes are *MYO7A* (MIM *#*276903), USH2A (MIM #608400), *CDH23* (MIM #605516), *CLRN1* (MIM #606397), *PCDH15* (MIM #605514), *USH1G* (MIM #606943), and *CIB2* (MIM #605564). In general, *MYO7A* is the most frequent cause of USH type1 accounting for approximately 50% of cases [19, 20]. Although, studies have reported variants in *CRB1*, *PDE6A*, *PDE6B*, *RP1*, *TULP1, CLRN1, MERTK* and *CNGA1* causing non-syndromic RP and *MYO7A*, *USH2A*, *CDH23*, *USH1H*, *PCDH15* genes are responsible for USH phenotypes in families of Pakistani origin [8, 21–28]. Among these genes, the most causative USH genes are *MYO7A* and *CDH23* having relative abundance of 42.9% and 28.6% respectively [22]. There are many Pakistani families with non-syndromic RP and USH phenotypes, however, the underlying genetic cause remains undetermined.

In Pakistan, geographical constraints and marriage patterns within communities may give rise to genetic isolates in which an increased frequency of certain disease-associated founder variants may occur [29–32]. Knowledge of the specific spectrum and frequency of disease-associated gene variants within different regions is fundamental to the development of effective and more tailored diagnostic genetic testing strategies, targeting variants relevant to a particular population. As part of an ongoing international collaborations, we are conducting genomic studies to define the likely specific molecular cause of disease in a group of families from Pakistan with a preliminary clinical diagnosis of retinal dystrophies. In current study, four consanguineous Pakistani families were analyzed to elucidate the genetic basis of non-syndromic RP and USH.

## Methodology

### Subjects

The current study was approved by the Ethical Committee and Advanced Studies and Research Board (ASRB), KUST, Pakistan. Families with a preliminary clinical diagnosis of non-syndromic RP and USH were referred for the genetic analysis by the collaborating local ophthalmologists between September, 2021 and March, 2022 in Khyber Pakhtunkhwa region of Pakistan. Three families had non-syndromic RP and one family had USH phenotypes. The study was conducted in accordance with the Declaration of Helsinki, informed written consent was obtained from the participating members of the families and from the parents of minor children. Family histories and demographic characteristics of the investigated families were recorded, and pedigrees were drawn. All the families practice consanguineous marriages (Table 1). Disease associated features of probands and other affected individuals in families were documented. Blood samples were collected from affected and normal members of the families.

### Genetic analysis

Genomic DNA was extracted from the blood samples via the ReliaPrep™ kit (Blood gDNA Miniprep System, Promega) and MagMAX DNA Multi-Sample Ultra 2.0 Kit using automated system KingFisher Apex (thermos scientific) and standard Phenol-Chloroform method. Whole exome sequencing (WES) was performed on the probands’ genomic DNA (III:1 in family-1, III:4 in family-2, III:1 in family-3 and II:6 in family-4) to find putative pathogenic variants. Coding regions were captured with HiSeq 4000 instrument (Illumina) with an average coverage of 100–120X at each nucleotide position and Twist human core exome kit (Twist Bioscience) through CeGat GmbH (Tübingen, Germany). Novoalign software (V3.08.00; Novocraft Technologies) was used to align the captured reads to reference human genome (build hg19). Picard version 2.140 SNAPSHOT used to remove duplicate reads. Genome analysis toolkit GATK (v3.8) was used for single base quality score and recalibration. Copy number variant (CNV) was detected with Exome Depth (v2.1). Happlotype Caller was used to generate Variant Call Format (VCF) file contain all genes variants [33]. AutoMap was used for homozygosity mapping [34]. Allele frequencies were detected using gnomAD database (https://gnomad.broadinstitute.org/), and conservation of variants was performed by using GERP [35]. Sanger sequencing was performed to likely pathogenic variant in all the recruited individuals. Single nucleotide polymorphisms (SNPs) were filtered-out, if present in the 1000 Genomes Project (www.1000genomes.org) or the Single Nucleotide Polymorphism Database (dbSNP; NCBI) to select the candidate genes. The variant-specific primers were designed using online available primer3 plus tool (https://www.primer3plus) to find the segregation of disease-causing variants in the family members. The DNA samples were amplified using standard PCR programing and protocols. The amplified products were sequenced by Source BioScience LifeSciences (https://www.sourcebioscience.com/) and Microsynth AG (https://www.microsynth.com). The segregating genetic variants were analyzed using SIFT (https://sift.edu.sg) and Mutation Taster (www.mutationtaster.org) to predict the impact of the sequence variations on encoded proteins’ function. The protein-protein interaction data was assembled from KEGG pathways (https://www.kegg/pathway) and String (https://string-db.org/) databases. In addition, the Varsome (https://varsome.com) online tool was used for variants classification according to the American College of Medical Genetics and Genomics (ACMG).

To compare and correlate the segregating genetic variants with phenotype, all reported variants were retrieved from HGMD (http://www.hgmd), OMIM (https://www.omim), PubMed (https://www.pubmed), ClinVar (https://www.ncbi.nlm.nih.gov/clinvar/) databases.

## Result

### Families description

In the present study, 4 consanguineous families were analyzed from different regions of Khyber Pakhtunkhwa (Table 1). In total 42 individuals in four families were investigated, among them 20 were affected individuals including 6 in both family 1 and 2, 5 in family 3 and 3 in family 4. Three families (Family 2, 3 and 4) with non-syndromic RP had initial symptom of night blindness with variable age of onset whereas a family (Family 1) with USH phenotypes had profound congenital deafness, balance problems and age of onset of RP was in adolescence. Furthermore, maculopathy was observed in affected individuals of a family with RP (Family 2). In addition, nystagmus, cone dystrophy and complete blindness at the age of 20 year were observed in affected individuals of a family with RP (Family 3). In the same family, some affected members had mild symptoms of RP. The clinical signs observed on fundus of probands and affected individuals with retinal dystrophies are summarized in Table 1. Pedigrees analysis showed that all the recruited families had autosomal recessive pattern of disease inheritance due to consanguineous marriages.

**Table 1:** Clinical characteristics of patients in the investigated families

	Patient ID	Location (Ethnicity)	Age (years)	Onset Age (years)	Clinical Symptoms	Clinical Signs on Fundus	Retinal Dystrophy	Identified Variants	
**Family 1**	III:1	Waziristan (Pashtun)	32	12	Profound bilateral congenital deafness, postural instability, night blindness and decreased vision (progressive)	Bone spicules, attenuated retinal vessels & waxy pallor of the optic disc	USH1	*MYO7A* (c.487G > A) **Homozygous**	
**Family 2**	III:4	Hangu (Pashtun)	20	8	Night blindness	Bilateral cellophane maculopathy and bone spicule	RP	*MERTK* (c.2194 C > T) **Homozygous**	
**Family 3**	III:1	Kohat (Pashtun)	21	8	Decreased vision with progressive deterioration, night blindness and nystagmus, now blind	High frequency high amplitude nystagmus, peripheral bone spicule pigmentary retinopathy, attenuated retinal vessels and waxy pallor of the optic disc	RP	*TULP1* (c.238G > A) **Homozygous**	*PDE6C* (c.480delG) **Homozygous**
	III:2	Kohat (Pashtun)	18	14	Decreased vision with progressive deterioration and severe photophobia	Bilateral maculopathy (inherited macular dystrophy)	Cone Dystrophy	*TULP1* (c.238G > A) **Heterozygous**	*PDE6C* (c.480delG) **Homozygous**
	III:3	Kohat (Pashtun)	11	8	Decreased vision with progressive deterioration and night blindness	Peripheral bone spicule pigmentary retinopathy Attenuated retinal vessels Waxy pallor of the optic disc	RP	*TULP1* (c.238G > A) **Homozygous**	*PDE6C* (c.480delG) **Homozygous**
	III:4	Kohat (Pashtun)	12	8	Decreased vision and night blindness	Peripheral bone spicule pigmentary retinopathy (mild), attenuated retinal vessels and waxy pallor of the optic disc	RP	*TULP1* (c.238G > A) **Homozygous**	*PDE6C* (c.480delG) **Heterozygous**
	III:5	Kohat (Pashtun)	8	8	Decreased vision and night blindness	Peripheral bone spicule pigmentary retinopathy (mild), attenuated retinal vessels and waxy pallor of the optic disc	RP	*TULP1* (c.238G > A) **Homozygous**	*PDE6C* (c.480delG) **Heterozygous**
**Family 4**	II:6	Kohat (Pashtun)	23	10	Night blindness	Bone spicule Attenuated retinal vessels, waxy pallor of the optic disc	RP	*RHO* (c.448G > A) **Homozygous**	

### Genetic findings

Likely causative genetic variants were identified in all the four families (Family-1, 2, 3 and 4) (Table 2).

**Table 2:** Summary of the pathogenic variants identified in investigated families

Family #	Gene	Nucleotide Variant	Protein Variant	ACMJ classification	Allele Frequency	Reference
Family 1	*MYO7A*	c.487G > A	p.Gly163Arg	Pathogenic	0.000007	(38,39)
Family 2	*MERTK*	c.2194 C > T	p.Arg732Ter	Pathogenic	0.00	(45)
Family 3	*TULP1*	c.238 C > T	p.Gln80Ter	Pathogenic	0.00003	ClinVar (971453)
	*PDE6C*	c.480delG	p.Asn161ThrfsTer33	Pathogenic	0.00	Novel (Current Study)
Family 4	*RHO*	c.448G > A	p.Glu150Lys	Pathogenic	0.00004	(63)

A rare homozygous missense variant NM_000260.3:c.487G > A in exon 6 in the *MYO7A* gene was found segregating in family1 with USH phenotype. The *MYO7A* missense variant leads to the substitution of glycine with arginine at evolutionary conserved position at 163 (p.Gly163Arg) in protein. A rare homozygous missense variant NM_006343.3:c.2194 C > T in exon 17 in the *MERTK* gene was identified in family2 affected members who had both RP and maculopathy phenotypes. The *MERTK* variant is predicted to result in a premature stop codon (p.Arg732Ter) and expression of the aberrant transcript harboring truncating variant and its degradation by nonsense-mediated mRNA decay (NMD) has been predicted by in silico tools.

**Fig. 1:**
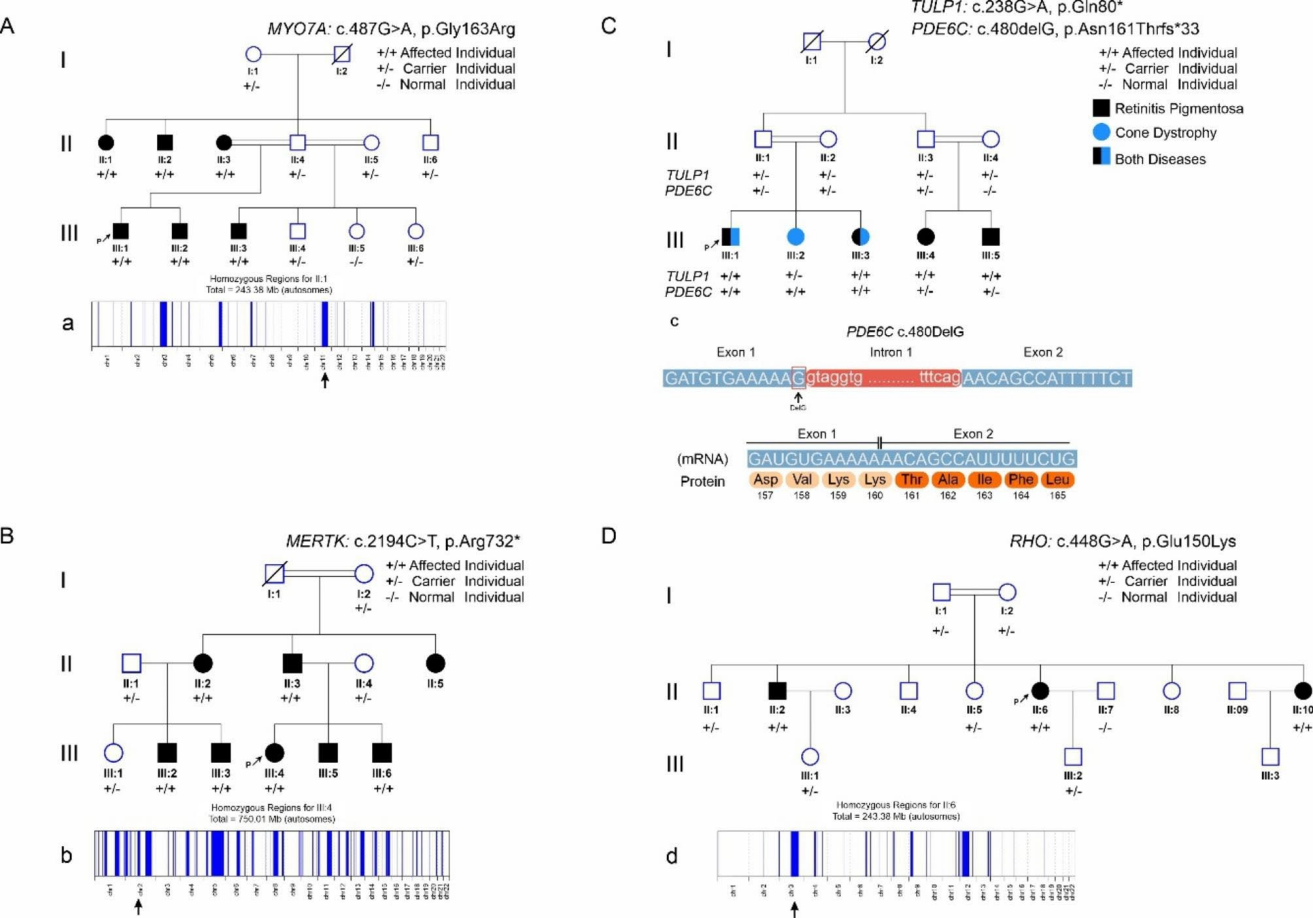
Families pedigrees and genetic findings: **A**. Pedigree of family 1 showing segregation of the identified variant in *MYO7A* gene, **a.** Homozygosity map of the proband (III:1), showing the runs of homozygosity as blue band. **B**. Pedigree of family 2 showing segregation of the identified variant in *MERTK* gene, **b.** Homozygosity map of the proband (III:4), showing the runs of homozygosity as blue band. **C.** Pedigree of family 3 showing segregation of the identified variants in *TULP1* and *PDE6C* genes. **c**. Schematic representation of the pathogenic *PDE6C* mutation (c.480delG) on the protein sequence predicted by in-silico tools. **D.** Pedigree of family 4 showing segregation of the identified variant in *RHO* gene **b**. Homozygosity map of the proband (II:6), showing the runs of homozygosity as blue band

A novel *PDE6C* frameshift deletion variant NM_006204.4:c.480delG in exon 1 and a rare *TULP1* nonsense variant NM_003322.6:c.238 C > T in exon 4, were observed to segregate with progressive cone dystrophy and non-syndromic RP respectively in family 3. Notably, nystagmus and complete blindness at the age of 20 years were found additional features in an individual who was detected homozygous for both *PDE6C* and *TULP1* mutant alleles. However, mild features of RP were observed in some individuals of the same family who were detected homozygous for only *TULP1* variant alleles and cone dystrophy was observed in an individual who was carrying only *PDE6C* variant alleles in homozygous state (Table 1). The *PDE6C* deletion variant (c.480delG) result in a frameshift followed by a premature stop codon (p.Asn161ThrfsTer33) whereas *TULP1* missense variant (c.238 C > T) also introduces a premature stop codon (p.Gln80Ter). The *PDE6C* variant c.480delG results in deletion of the last nucleotide in exon 1 therefore, some of the in-silico tools suggested that the variant (c.480delG) may result in broken wild type donor splice site (c.480 + 1delG) (Human Splicing Finder and Mutation Taster). However, a gain of splice donor site is also predicted by SpliceAI at 480 position suggesting normal splicing (Fig. 1c). In-silico analysis further revealed that the RNA transcripts with *PDE6C* and *TULP1* truncating variants are degraded by NMD.

A homozygous missense variant NM_000539.3:c.448G > A in exon 2 in the *RHO* gene was found segregating with non-syndromic RP phenotype in family4. The missense *RHO* variant (c.448G > A) leads to the substitution of glutamic acid with lysine at evolutionary conserved position 150 (p.Glu150Lys) in protein. Importantly, the identified *TULP1*, *MERTK* and *MYO7A* variants were first time found segregating with non-syndromic RP and USH phenotypes in Pakistani families. Interestingly, the segregation data revealed that among the 22 participating clinically normal members in the investigated families in which genotype-phenotype correlations were established, 20 (90.90%) individuals were detected carriers for a single variant allele and about 7 (31.8%) members in family 3 were detected carriers for two variant alleles.

Overall, the variant alleles were detected inside the long homozygous contigs exceeding 11 Mb in size in probands of family1, 2 and 4 (Supplementary data Tables). The sizes of total homozygous contigs of the families having variant in *MYO7A, MERTK* and *RHO* were 243.38 Mb, 750.01 Mb and 244.18 Mb respectively (Fig. 1).

## Discussion

Establishing molecular diagnosis in patients with RP is challenging in clinical practice due to the disease significant phenotypic and genetic heterogeneity [36]. Traditional approaches of individual gene screening are difficult, time consuming and don’t reveal the complete variant spectrum in RP patients. However, WES is currently the most efficient approach and widely used for identification of the molecular bases of heterogeneous genetic diseases [37]. In this study, causative variants in *TULP1*, *PDE6C*, *RHO*, *MERTK* and *MYO7A* genes were identified in all the 4 Pakistani families displayed either non-syndromic RP or USH phenotypes using WES approach. Studies have previously reported variants in *CRB1*, *PDE6A*, *PDE6B*, *RP1*, *TULP1, CLRN1, MERTK* and *CNGA1* causing non-syndromic RP and variants in *MYO7A*, *USH2A*, *CDH23*, *USH1H*, *PCDH15* genes causing USH in families of Pakistani origin [8, 21–28]. Importantly, delineating genetic basis of RP and USH in this study and in previous studies provides essential information for molecular diagnosis as well as for genetic counselling of extended families in order to reduce burden of these diseases in Pakistani population.

In family1 with USH phenotype, a rare homozygous missense variant (c.487G > A) in the *MYO7A* was found segregating with USH type 1 (USH1) phenotype, more specifically USH type IB (USH1B). This variant was first time reported by Sloan-Heggen et al. [38] in an affected individual with USH1 phenotype, later on this variant in compound heterozygous state was found to cause USH1 in an Algerian family [39], whereas, in homozygous state this variant caused non-syndromic hearing loss in a Turkish consanguineous family [40]. The findings of present and previous studies confirm that *MYO7A* is expressed in the retina and inner ear, thus variants in *MYO7A* are known to cause diseases affecting the retina and ear. *MYO7A* encodes myosin VIIA protein, a 2215 amino acids long protein in these organs where it acts on the transport processes through the cilia of the retinal photoreceptors or interacts with multiple proteins actin, harmonin, Sans, CDH23, and PCDH15 in stereocilia hair cells of the inner ear to form mechano-transduction complex, which is crucial for sound detection [41]. In the Human Gene Mutation Database (HGMD), more than 662 *MYO7A* variants have been listed till date that most commonly cause USH phenotypes. Among them, the variants such as p.Arg212Cys, p.Arg212His, p.Arg302His, p.Arg666Ter and p.Gln821Ter most frequently cause USH1 [42, 43]. In families of Pakistani origin, about 6.25% *MYO7A* variants have been reported to most likely cause congenital deafness and few variants cause USH1 [44]. The current study provides evidence of association of *MYO7A* variants with USH1. In addition, the reported high frequency of *MYO7A* variants causing congenital deafness in Pakistani population could be due to RP usual onset in adolescence and lack of clinical diagnostics facilities to make a diagnosis of RP in Pakistan.

In family2, a rarely reported nonsense *MERTK* variant c.2194 C > T caused non-syndromic RP and maculopathy. This variant was previously only reported by O’Sullivan et al. [45] in an RP patient from a British cohort. The *MERTK* gene encodes MER tyrosine kinase receptor that plays crucial role in phagocytosis of retinal pigment epithelium (RPE) [46]. This receptor exhibits four functional domains, such as immunoglobulin-like, fibronectin-like, transmembrane-like, and tyrosine kinase-like domains [47]. The MERTK receptor in retina is involved in the RPE phagocytosis in the inner segment to re-new the photoreceptor outer segments [48]. TULP1, Gas6 and protein S are the ligands that binds to MERTK receptor and activates the receptor upon phosphorylation of its kinase domain, that in turn activates the down-cascade for the phagocytosis. Previously reported MERTK variants (p.Ser627Ter, p.Arg651Ter, p.Asp487LeufsTer57, p.Trp131Ter, p.Ile103AsnfsTer4) were found to disturb phagocytosis pathway in the photoreceptor cells and consequently cause RP in consanguineous families [49–51]. However, *MERTK* variants in compound heterozygous state have been reported to cause arRP in multiple siblings of a non-consanguineous family as well [52]. The *MERTK* truncating variant p.Arg732Ter detected in current study is a loss of function variant due to the degradation of expressed aberrant transcript by NMD [53] that may lead to retinal dystrophies.

In family3, a novel frameshift deletion *PDE6C* variant (c.480delG, p.Asn161thrfs*33) and a rare nonsense *TULP1* variant (c. 238 C > T, p.Gln80Ter) independent segregated with progressive cone dystrophy and non-syndromic RP respectively. Importantly, the genotype-phenotype correlations have been established for both types of retinal dystrophies in this family. Sequence variants in *PDE6C* have been reported previously to cause autosomal recessive cone dystrophy [54–56]. In cone dystrophy, cone cells in retina are affected causing decrease in visual acuity and increased sensitivity to light. PDE6C protein is expressed in cone photoreceptors and plays important role in the rapid decrease of intracellular cGMP levels in the outer segment. Normally, PDE6C protein is known to be directly involved in phototransduction pathway inside cone cells (UniProtKB-P51160). In current study, the identified frameshift deletion (p.Asn161ThrfsTer33) in the *PDE6C* has been predicted as a loss of function variant due to the degradation of expressed aberrant transcript by NMD [53] that can cause progressive cone dystrophy. In addition, at same position another pathogenic missense variant (c.480G > T, p.Lys160Asn) reported in the ClinVar database (ClinVar; VCV001213877.1) causes Achromatopsia, a type of stationary cone dystrophy usually appears in childhood. This can be explained by the fact that the *PDE6C* variants identified in previous and present studies resulted in a change at an evolutionarily conserved position that severely affected protein’s function and consequently caused cone degeneration, a common feature in both stationary and progressive cone dystrophy.

Although, cone dystrophy associated *PDE6C* variant independently segregated from RP associated *TULP1* variant in family 3, however, nystagmus and complete blindness at the age of 20 years were observed in family members who carried both *PDE6C* and *TULP1* variants. In addition, mild features of RP were observed in individuals who were carried only *TULP1* variant. Previously, *TULP1* variants have commonly been found associated with non-syndromic RP [57, 58]. As the Tub Like protein 1 is expressed from *TULP1* gene in the rod and cone photoreceptor cells in retina and is involved in development of photoreceptor synapses as well as rhodopsin transportation from its site of synthesis in the inner segment of photoreceptor cells through the connecting cilium to the outer segment [59]. Therefore, most of the identified *TULP1* variants have been described to affect normal functions of the TULP1 protein in retinal cells and consequently cause RP. Moreover, association of *TULP1* variants with arRP has already been established [24, 57, 58, 60–62] and it was estimated that *TULP1* variants account for 2% of arRP cases [63]. The identification of causal variants in a Pakistani consanguineous family in present study reaffirmed the role of *TULP1* in the pathogenesis of arRP.

In family4, a missense *RHO* variant (c.448G > A; p.Glu150Lys) was identified to cause non-syndromic RP phenotype. This variant has been previously reported in two unrelated consanguineous Pakistani families [64]. Identification of recurrent *RHO* variant (p.Glu150Lys) will aid in establishing its founder effect in population of Pakistan and should be suggestively screened in RP susceptible families for premarital counseling. Although, the *RHO* variants commonly cause adRP and rarely cause arRP [65–68], however, in both cases role of RHO is important for normal functions of rod cells in retina. *RHO* gene encodes rhodopsin, a light-detecting G-protein-coupled receptor that plays a vital role in phototransduction and rod photoreceptor cell health. Rhodopsin consists of four specialized domains: the cytoplasmic, intradiscal, ligand-binding, and transmembrane domains. Each domain assists in the maintenance of either the Rhodopsin proper structure and its trafficking through the cell or its vital role in the phototransduction. The cytoplasmic C-terminal domain of Rhodopsin is crucial in regulation of membrane trafficking and its interactions with other proteins in the phototransduction cascade [69, 70]. It has been elucidated that sequence variations in this domain lead to retinal degenerations such as adRP and arRP [71, 72]. The *RHO* variant (p.Glu150Lys) identified in current study lies in the cytoplasmic domain of Rhodopsin that affects its signaling activity as a G-protein-coupled receptor and consequential aberrant trafficking as well as initiation of retinal degeneration usually lead to RP.

The probands in investigated families were consanguineous offspring who have elevated levels of homozygosity. Autozygous stretches within their genome were detected which likely harbored loss of function variants and resulted in complete inactivation or dysfunction of genes. The identified *MYO7A, MERTK* and *RHO* variants in the current study lie within regions of homozygosity (41 Mb, 11 Mb and 62 Mb respectively). Therefore, in current study consanguinity and homozygosity were detected risk of arRP and USH in Pakistani families. In addition, majority (90.90%) of clinically normal members in the investigated families were detected carriers for a single variant allele and few individuals were detected carriers for two variant alleles in a family in which both *PDE6C* and *TULP1* variants segregated independently. The high rate of carrier detection in investigated consanguineous families revealed that those individuals are at increased risk of transmitting the diseases to future generations.

Establishing the diagnosis for retinal dystrophies is quite challenging due to their high genetic heterogeneity and overlapping clinical presentations. These factors collectively make it extremely difficult to establish an accurate diagnosis based on clinical presentation alone in a country like Pakistan, where many families reside in highly remote rural regions and have limited access to primary healthcare as well as ophthalmic services. In addition, there is also limited availability of specific and expensive diagnostic investigations such as fundoscopy, electroretinography and optical coherence tomography that are required to assess ocular status in retinal dystrophies. It is worth mentioning here that application of modern genomic technologies enabled us to establish an accurate molecular diagnosis in investigated families with retinal dystrophies and has also facilitated informed genetic counselling.

In summary, the current study highlights the importance of next generation sequencing approach in finding the underlying genetic causes of RP and USH in Pakistani families. In addition, molecular genetic diagnosis was successfully established in all the four families. Unexpected genotype-phenotype correlations were identified in some families that confirmed association of RP with neurological and retinal defects. The current study provides substantial evidence that consanguinity and autozygosity increased risk for genetic diseases in our population. Furthermore, the carrier frequency for retinal dystrophies was found alarmingly high among phenotypically normal individuals in investigated Pakistani families.

## Electronic supplementary material

Below is the link to the electronic supplementary material.

Supplementary Material 1

## Data Availability

The datasets generated and/or analyzed during the current study are available in the ClinVar repository, with the accession numbers i.e. SCV002599429, SCV002600101, SCV002600102, SCV002600103 and SCV002600104.
